# Effect of dexamethasone on intraoperative remifentanil dose in total knee arthroplasty surgery under general anaesthesia

**DOI:** 10.1111/aas.14118

**Published:** 2022-08-04

**Authors:** Maria Gantzel, Kasper Smidt Gasbjerg, Daniel Hägi‐Pedersen, Christian Sylvest Meyhoff, Markus Harboe Olsen, Ole Mathiesen, Janus Christian Jakobsen, Troels Haxholdt Lunn

**Affiliations:** ^1^ Department of Anaesthesia and Intensive Care Copenhagen University Hospital—Bispebjerg and Frederiksberg Copenhagen Denmark; ^2^ Department of Anaesthesiology Research Centre of Anaesthesiology and Intensive Care Medicine, Næstved‐Slagelse‐Ringsted Hospitals Slagelse Denmark; ^3^ Department of Clinical Medicine Copenhagen University Copenhagen Denmark; ^4^ Copenhagen Trial Unit, Centre for Clinical Intervention Research Copenhagen University Hospital—Rigshospitalet Copenhagen Denmark; ^5^ Department of Neuroanaesthesiology, The Neuroscience Centre Copenhagen University Hospital—Rigshospitalet Copenhagen Denmark; ^6^ Department of Anaesthesiology Centre for Anaesthesiological Research, Zealand University Hospital Køge Denmark; ^7^ The Faculty of Health Sciences, Department of Regional Health Research University of Southern Denmark Odense Denmark

**Keywords:** alloplastic surgery, dexamethasone, glucocorticoid, intraoperative pain, intraoperative remifentanil, remifentanil anaesthesia, total knee arthroplasty, total knee replacement

## Abstract

**Background:**

The effects of glucocorticoids may include both genomic and rapid nongenomic effects. The potential rapid analgesic effect during surgery has not previously been investigated. We aimed to explore the effect of dexamethasone on intraoperative infusion rate of remifentanil in patients undergoing total knee arthroplasty (TKA) surgery under general anaesthesia.

**Methods:**

In this post hoc subgroup analysis, we included patients randomised in the DEX‐2‐TKA trial, who were operated under total intravenous anaesthesia with remifentanil and propofol. Trial medication, intravenous dexamethasone 24 mg or placebo, was administered immediately after anaesthesia onset. The primary outcome was the median weight‐corrected infusion rate of remifentanil during surgery. Secondary outcomes included median weight‐corrected infusion rate of propofol, median intraoperative bispectral index and time spent in the post‐anaesthesia care unit.

**Results:**

Eighty‐seven patients were included in the analysis of the primary outcome. A significantly higher remifentanil infusion rate was observed in the dexamethasone group compared with the placebo group, *p =* .02. None of the secondary outcomes resulted in statistically significant differences between groups.

**Conclusion:**

This explorative post hoc analysis of the randomised DEX‐2‐TKA trail showed that patients undergoing TKA surgery under general anaesthesia and who received dexamethasone seemed to have a higher remifentanil infusion rate compared with patients who received placebo. The clinical implications of the potentially increased remifentanil infusion rate need to be validated and explored further.

**Clinical trial registration:**

ClinicalTrials.gov Identifier: NCT05002361 (12 August 2021).

**Editorial Comment**

Pre‐operative dexamethasone has been used to try to reduce early post‐operative pain. In this post hoc analysis from trial data which found this post‐operative analgesic effect, even earlier and intraoperative analgesic effects were explored. The findings included higher received doses of intraoperative remifentanil associated with dexamethasone pre‐treatment, which was unexpected.

## 1 INTRODUCTION

Glucocorticoids have anti‐inflammatory and immunosuppressive effects and are recommended for post‐operative nausea and vomiting (PONV) prophylaxis.^1^, ^2^, ^3^ Recently, the randomised, blinded, multicentre DEX‐2‐TKA trial showed a reduction in pain intensity and morphine consumption the first 48 h after surgery in patients undergoing total knee arthroplasty (TKA) given one and two doses of 24 mg dexamethasone compared with placebo.^4^

Glucocorticoids inhibit both the early and the late mechanisms that contribute to the inflammatory response.^5^ This includes direct and indirect effects on gene expression (onset within hours) and nongenomic effects (onset within minutes) both with analgesic and anti‐hyperalgesic effects.^5^, ^6^, ^7^ The potential rapid onset of the analgesic effects during surgery has, to our knowledge, not previously been investigated.

For general anaesthesia, remifentanil, a synthetic short acting μ‐1 opioid receptor agonist, is often used. Remifentanil provides strong analgesia with a rapid onset and a short halftime,^8^ but may also be associated with adverse effects such as opioid induced hyperalgesia, opioid tolerance, increased post‐operative opioid consumption, post‐anaesthetic shivering, hypotension, bradycardia and muscle rigidity.^9^, ^10^, ^11^ Thus, the lowest possible dose of remifentanil for adequate anaesthesia should be applied.

In this post hoc subgroup analysis of the DEX‐2‐TKA trial, we aimed to explore the hypothesis that dexamethasone administered shortly before surgery lowers the intraoperative infusion rate of remifentanil compared with placebo. Secondarily, we aimed to explore the effects on the intraoperative infusion rate of propofol, anaesthesia depth and time spent in the post‐anaesthesia care unit (PACU).

## 2 METHODS

### 2.1 DEX‐2‐TKA study design and trial procedures

DEX‐2‐TKA was a randomised, blinded, multicentre trial including participants undergoing primary TKA, conducted to investigate the effects of dexamethasone on morphine consumption, levels of post‐operative pain and harm.^12^ The trial was approved by the Regional Committee on Health Research Ethics, Region Zealand (SJ‐695; 07 May 2018), the Danish Medicines Agency (EudraCT‐number 2018‐001099‐30; 25 May 2018) and the Danish Data Protection Agency (REG‐034‐2018; 14 August 2018) and was monitored by the Good Clinical Practice Units at Copenhagen and Odense University Hospitals, Denmark. The methodology has previously been described in detail in the published protocol,^12^ the statistical analysis plan^13^ and the main result publication.^4^ In short, the trial was conducted at one private and four public Danish hospitals. Patients were randomised into one of three groups receiving either: dexamethasone + placebo, dexamethasone + dexamethasone or placebo + placebo in a 1:1:1 ratio. The first dose of trial medication (intravenous dexamethasone 24 mg or placebo) was administered immediately after onset of anaesthesia. Twenty‐four hours after end of surgery, the second dose (intravenous dexamethasone 24 mg or placebo) was administered. Patients received either spinal anaesthesia or general anaesthesia (where remifentanil and propofol were preferred). Propofol and remifentanil were titrated to achieve a temporary comatose patient eligible for surgery, with pulse and blood pressure within the normal range and ceased ciliary reflex.^14^, ^15^ Before end of surgery, all patients received intravenous ondansetron 4 mg. For patients operated under general anaesthesia, intravenous sufentanil (0.3 μg kg^−1^) was administered at the end of surgery. All participants received a protocolled non‐opioid analgesic pain alleviation regime comprised of oral paracetamol 1 g and ibuprofen 400 mg given 1 h before and every 6 h after surgery, and the surgeon administered local infiltration analgesia, 150 ml ropivacaine 1.444 mg ml^−1^, intraoperatively according to a standardised regimen.

### 2.2 Post hoc study design

The present study is an explorative post hoc subgroup analysis including patients randomised in the DEX‐2‐TKA trial, who were operated under general anaesthesia. Patients, who did not receive general anaesthesia with remifentanil and propofol and patients with missing anaesthesia journal were excluded.

As the two original groups from the DEX‐2‐TKA trial, who received dexamethasone shortly before surgery, were identical at the time of outcome assessment in the present study, they were merged to one and compared with placebo. Despite being a post hoc analysis, outcomes and statistical analysis plan were defined, and the trial independently registered with clinicaltrials.gov (identifier: NCT05002361, 12 August 2021) prior to data analyses and unblinding of the post hoc study population.

### 2.3 Outcomes measures

The primary outcome was the median weight‐corrected infusion rate of remifentanil (total amount of intraoperative remifentanil adjusted for body weight and duration of surgery) from initial surgical incision until last suture (μg kg^−1^ h^−1^).

The secondary outcomes were the median weight‐corrected infusion rate of propofol from initial surgical incision until last suture (mg kg h^−1^); the median intraoperative bispectral index (BIS) from initial surgical incision until last suture (only for patients at Næstved Hospital where it is a standard measure) and the median time spent in PACU (from arrival at PACU until transfer to the orthopaedic ward, in minutes). For all outcomes, comparisons between the dexamethasone group and the placebo group were conducted.

### 2.4 Data collection

To collect data from the participating hospitals, Sundhedsplatformen (EPIC, Verona, WI, USA) was used at Bispebjerg Hospital, Næstved‐Slagelse‐Ringsted Hospitals and Køge Hospital. For Gildhøj Private Hospital and Odense University Hospital, data were collected from handwritten anaesthesia reports. Data on propofol and remifentanil were collected from initial surgical incision till last suture. If anaesthesia was converted from spinal anaesthesia to general anaesthesia after surgical incision, data were collected from airway management till last suture. Data on infusion rate and exact time for changes were collected. These data were used to calculate the total amount of remifentanil and propofol using the following concentrations: propofol 10 mg ml^−1^ and remifentanil 50 or 60 μg ml^−1^, and corrected for body weight and duration of surgery. Body weight was collected from the electronic medical record as either measured or patient reported body weight before surgery. Data on time spent in PACU were also collected from anaesthesia reports either handwritten or from EPIC. Time spent in PACU was calculated from arrival till departure from PACU. BIS data were collected from EPIC only on patients from Næstved Hospital, where BIS is used as standard measure during surgery. BIS data were registered every 5 min from initial surgical incision till last suture.

### 2.5 Statistical methods

This post hoc subgroup analysis was explorative by nature, and we used a significance level of *p* ≤ .05. The analyses were carried out for all patients.

The statistical analyses were planned and made public after primary DEX‐2‐TKA data collection but before data analyses of this post hoc study. Analyses were conducted blinded to allocation. Testing assumptions for the pre‐planned linear regression analyses showed none of the outcomes to be normally distributed. Therefore, all outcomes were analysed using non‐parametric statistical methods, the same used in the DEX‐2‐TKA trail.^4^ Van Elteren test was used for data where adjustment for site was needed. Wilcoxon rank sum test without continuity correction was used for BIS where data were only available for one site. Results were presented as median, interquartile range (IQR), Hodges–Lehmann median difference (HLMD) with corresponding 95% confidence intervals (CI) and *p*‐values. Assessments of underlying statistical assumptions were performed following the recommendations by Nørskov et al.^16^

Two statisticians (J. C. J. and M. H. O.) independently prepared a statistical report, which were compared for any discrepancies. The discrepancies were logged and any changes for the final report were described. The final report, the two original reports and the discrepancies log are attached as Supplemental Material.

## 3 RESULTS

**FIGURE 1:**
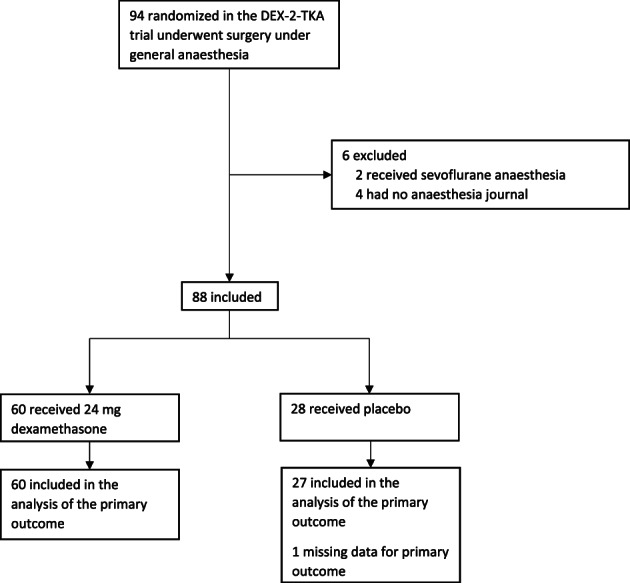
Exclusion and primary outcome population

In the DEX‐2‐TKA trial 485 patients were randomised, hereof 94 (19.4%) patients underwent surgery under general anaesthesia. Six patients were excluded due to missing anaesthesia journal or use of sevoflurane anaesthesia. Thus, a total of 88 patients were included, and 87 patients were included in the analysis of the primary outcome (1 missing due to lack of infusion data from the propofol and remifentanil pumps) (Figure 1).

There were minor differences in baseline characteristics between groups in the proportion of male sex, Type 2 diabetes and daily use of analgesics in the last month (Table 1).

**TABLE 1:** Baseline and perioperative characteristics

Intervention group	Dexamethasone (*N* = 60)	Placebo (*N* = 28)
*Baseline characteristics*		
Age—year, mean (SD)	69 (±10.0)	69 (±8.8)
Male—sex, no. (%)	23 (38%)	12 (43%)
Female—sex, no. (%)	37 (62%)	16 (57%)
American Society of Anaesthesiologists Physical Status—no. (%)		
Healthy	5 (8%)	2 (7%)
Mild systemic disease	45 (75%)	21 (75%)
Severe systemic disease	10 (17%)	5 (18%)
Body mass index, kg (m^2^)^−1^—mean (SD)	30 (±4.6)	31 (±4.5)
Diabetes Type 2—no. (%)	6 (10%)	4 (14%)
Insulin treated	0/6 (0%)	0/4 (0%)
Other diabetic treatments	5/6 (83%)	3/4 (75%)
Prior daily use (last month) of analgesic medication—no. (%)		
Paracetamol (acetaminophen)	29 (48%)	13 (46%)
NSAID	17 (28%)	7 (25%)
Opioids (max 30 mg morphine equivalents/day)	4 (7%)	1 (4%)
*Perioperative characteristics*		
Duration of surgery, min—median (IQR)	64 (57–71)	68 (58–78)
Type of knee arthroplasty—no. (%)		
Cemented	1 (1.7%)	0 (0%)
Cement less	26 (43.3%)	14 (50.0%)
Hybrid	33 (55.0%)	14 (50.0%)
Type of anaesthesia—no. (%)		
Planned total intravenous general anaesthesia	54 (90.0%)	24 (85.7%)
Conversion of spinal to total intravenous general anaesthesia	6 (10.0%)	4 (14.3%)
Amount of sufentanil, μg—mean (SD)	25 (±8.3)	24 (±12.0)
Blood loss, ml—median (IQR)^a^	150 (100–200)	150 (39–200)
Administration of 4 mg ondansetron PONV‐prophylaxis—no. (%)	56 (93.3%)	27 (96.4%)
Administration of local infiltration analgesia—no. (%)	59 (98.3%)	28 (100%)

Abbreviations: IQR, interquartile range; NSAID, nonsteroidal anti‐inflammatory drugs; PONV, post‐operative nausea and vomiting.

^a^ Intraoperative blood loss was registered at the end of surgery comprising blood in the suction bottle and gauze.

**FIGURE 2:**
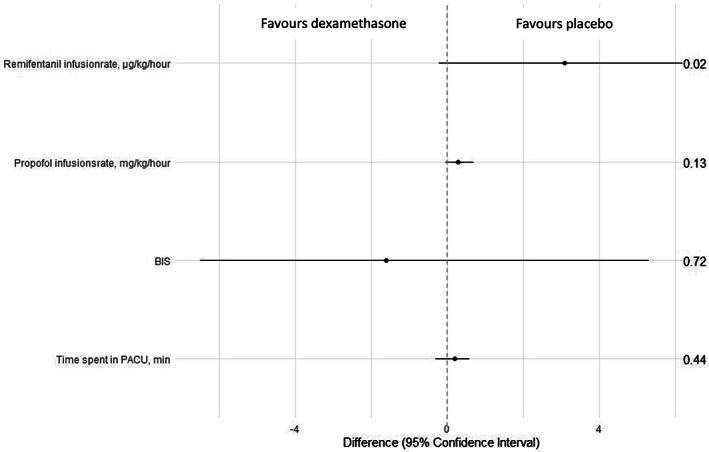
Illustration of interventions

A significantly higher remifentanil infusion rate was observed for patients in the dexamethasone group versus the placebo group; median: 23; IQR: 19–28 μg kg^−1^ h^−1^ versus median: 20; IQR: 16–25 μg kg^−1^ h^−1^; HLMD 3.1; 95% CI −0.2 to 6.2 μg kg^−1^ h^−1^; *p =* .02 (Table 2, Figure 2).

**TABLE 2:** Primary outcome: remifentanil infusion rate

Intervention group	Dexamethasone (*N* = 60)	Placebo (*N* = 27)
Remifentanil infusion rate, μg kg^−1^ h^−1^—median (IQR)	23 (19–28)	20 (16–25)
Difference (95% CI), μg kg^−1^ h^−1^	3.1 (−0.2 to 6.2), *p* = .02	

*Note*: Difference between medians is calculated using Hodges–Lehmann estimator; *p*‐value has been calculated using van Elteren test.

Abbreviations: CI, confidence interval; IQR, interquartile range.

None of the secondary outcomes resulted in statistically significant differences between groups (Table 3, Figure 2).

**TABLE 3:** Secondary outcomes: propofol infusion rate, bispectral index and time spent in PACU

Intervention group	Dexamethasone (*N* = 60)	Placebo (*N* = 27)
Propofol infusion rate, mg kg^−1^ h^−1^—median (IQR)	4 (3–5)	4 (3–4)
Difference (95% CI), mg kg^−1^ h^−1^	0.3 (−0.04 to 0.7), *p* = .13	
	*N* = 18	*N* = 11
BIS—median (IQR)	48 (41–55)	45 (43–54)
Difference (95% CI)	−1.6 (−6.5 to 5.3), *p* = .72	
	*N* = 59	*N* = 28
PACU time, min—median (IQR)	135 (90–176)	111 (92–181)
Difference (95% CI), min	0.2 (−0.3 to 0.6), *p* = .44	

*Note*: Differences between medians are calculated using Hodges–Lehmann estimator; *p*‐values have been calculated using van Elteren test.

Abbreviations: BIS, Bispectral index; CI, confidence interval; IQR, interquartile range; PACU, post‐anaesthesia care unit.

**FIGURE 3:**
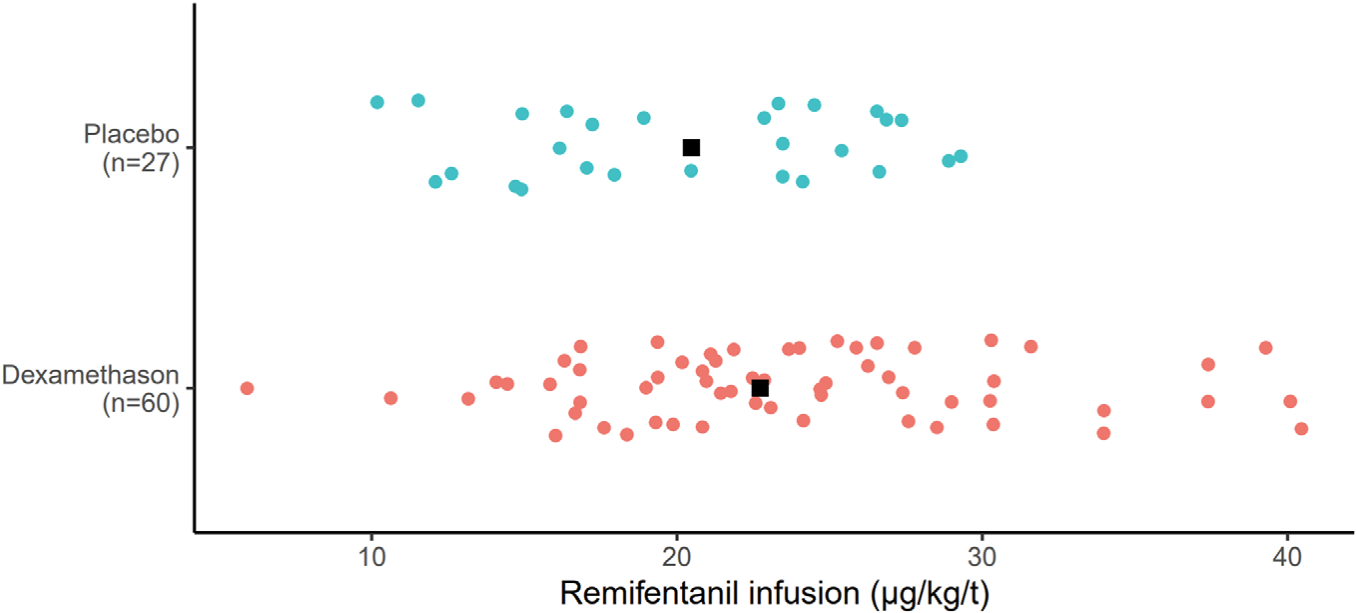
Histogram presenting each patient in the placebo and dexamethasone group on the *y*‐axis and the corresponding remifentanil infusion rate on the *x*‐axis

Furthermore, a histogram has been made presenting the placebo group and the dexamethasone group on the *y*‐axis with each patient represented as a dot corresponding to the remifentanil infusion rate on the *x*‐axis (Figure 3 **)**.

## 4 DISCUSSION

This study found a higher remifentanil infusion rate during TKA surgery in patients who received dexamethasone shortly after general anaesthesia induction compared with patients who received placebo. No significant differences were found in propofol infusion rate, BIS and time spent in PACU.

These exploratory results contradict our hypothesis of an analgesic sparing effect of dexamethasone.^17^ However, the magnitude of the observed difference in remifentanil infusion rate was small. The difference corresponds to a median difference of 248 μg (≈ 5 ml remifentanil of standard concentration) for a standard patient of 80 kg h^−1^. Thus, the clinical relevance should be explored further. The finding might also be random (Type 1 error) due to the post hoc design, as discussed under limitations. However, since no other studies have investigated the potential effect of dexamethasone given shortly before surgery on intraoperative remifentanil infusion rate, potential factors influencing on the result should be considered, as discussed below.

Remifentanil is a well‐known analgesic drug with anaesthetic properties that makes it suitable for total intravenous anaesthesia.^9^ Because of the analgesic properties, the infusion rate of remifentanil is often regulated when surgical pain stimuli appear in order to prevent pain, awareness and sympathetic activation. Scott et al. showed that loss of response to pain occurred at 4.4 μg ml^−1^ in patients receiving only propofol, and at 2.7 μg ml^−1^ in patients receiving propofol plus remifentanil.^18^ A time‐ and body weight‐corrected remifentanil infusion rate may therefore be used as a surrogate marker for the level of intraoperative pain.

Our hypothesis was based on the assumption that a fast‐inserting analgesic effect of dexamethasone exists. This cannot be excluded by our findings. However, our primary finding indicates that the potential fast‐inserting analgesic effect of dexamethasone is not measurable by the intraoperative use of remifentanil.

Dexamethasone may, however, also have other effects potentially explaining our findings. This includes hemodynamic effects such as hypertension.^19^ These hemodynamic effects of dexamethasone have been suggested to reduce mortality in patients with septic shock as treatment with dexamethasone may be associated with shock reversal and more vasopressor‐free days.^20^, ^21^ However, dexamethasone has the lowest (if any) mineralocorticoid effect of commonly used glucocorticoids and was therefore chosen in the DEX‐2‐TKA trail.^22^, ^23^ Dexamethasone may also induce mood symptoms such as euphoria or hypomania.^24^, ^25^ Glucocorticoid effects on the central nervous system appear to be dose‐dependent, thus increasing the risk of hypomania with high‐dose dexamethasone.^24^, ^25^ Therefore, questions could be asked on whether these phenomena might affect the dose of remifentanil needed to avoid awareness.

As no valid single measure of intraoperative pain exists, the anaesthetist depends on clinical parameters to titrate remifentanil to adequate anaesthesia. In clinical practice and in the present study it was based on an assessment by the anaesthetist.^14^ Thus, it may be speculated that the higher remifentanil infusion rate observed in the dexamethasone group may be explained by increased blood pressure or hypomania, handled by the anaesthetist with increased remifentanil infusion rate.

### 4.1 Limitations and strengths

The strength of this study is the high‐quality data derived from the randomised, blinded, multicentre DEX‐2‐TKA trial with only few missing data.^4^ The analyses were performed blinded by two independent statisticians, preparing two separate statistical reports and afterwards comparing for discrepancies in a third report.

The study also has limitations. It is a post hoc analysis with inherit Type 1 error risk for the significant primary finding, and results are by nature exploratory and hypothesis generating. However, despite being a post hoc analysis, outcomes and statistical analysis plan were pre‐defined (but after data collection), and the trial independently registered with clinicaltrials.gov prior to data analyses and unblinding.^17^ Moreover, the study population was relatively small and especially data on BIS was limited. Thus, the lack of statistical evidence for a difference regarding insignificant secondary outcomes might be due to lack of power (Type 2 error). Furthermore, the adjustment of infusion rates of remifentanil and propofol are subjectively based on clinical assessment by the anaesthetist as in clinical practice but is also a study limitation. Moreover, the infusion rates were corrected for body weight and time of surgery, however, other confounding factors not accounted for might also influence on the infusion rates and results. Finally, the 95% CI of the remifentanil infusion rate includes 0. This indicates that the result is insignificant. However, Hodges–Lehman cannot stratify for site like the Van Elteren test, which explains the discrepancies in the result of the *p*‐value and 95% CI for the primary outcome. We pre‐defined to analyse data using the van Elteren test, and that result should be considered our primary result. However, whether we identified a statistically significant difference depended on the chosen statistical method, which should be considered when interpreting our results.

In conclusion, this explorative post hoc analysis of the randomised DEX‐2‐TKA trial showed that patients undergoing TKA under general anaesthesia with remifentanil and propofol and who received dexamethasone shortly before surgery, seemed to have a higher remifentanil infusion rate compared to patients who received placebo, and thereby contradicting our primary hypothesis. No significant differences were found in propofol infusion rate, BIS or time spent in PACU. The clinical implications of the potentially increased remifentanil infusion rate need to be validated and explored further.

## AUTHOR CONTRIBUTIONS

**Maria Gantzel**: Study design, statistical analysis, writing the manuscript, data interpretation, editing of the manuscript and are responsible for data collection. **Troels Haxholdt Lunn**: Study design, writing the manuscript, data interpretation and editing of the manuscript. **Kasper Smidt Gasbjerg**: Study design, writing the manuscript, data interpretation and editing of the manuscript. **Janus Christian Jakobse**: Study design, data interpretation, editing of the manuscript and are responsible for data analysis of the primary and secondary outcome. **Markus Harboe Olse**: Study design, data interpretation, editing of the manuscript and are responsible for data analysis of the primary and secondary outcome. **Ole Mathiesen**: Study design and editing of the manuscript. **Christian Sylvest Meyhoff**: Study design and editing of the manuscript. **Daniel Hägi‐Pedersen**: Study design and editing of the manuscript. The corresponding author attests that all listed authors meet authorship criteria and that no others meeting the criteria have been omitted.

## FUNDING INFORMATION

This work used data from the DEX‐2‐TKA trial, which was supported by Næstved, Slagelse and Ringsted Hospitals' Research Fund and received support from the Department of Anaesthesiology, Næstved, Slagelse and Ringsted Hospitals, Denmark. The funder of the study had no role in the design and conduct of the study; collection, management, analysis and interpretation of the data; preparation, review or approval of the manuscript; and decision to submit the manuscript for publication.

## CONFLICTS OF INTEREST

Ole Mathiesen is an associate editor at Acta Anaesthesiologica Scandinavica. Christian Sylvest Meyhoff is an associate editor at Acta Anaesthesiologica Scandinavica. Christian Sylvest Meyhoff has co‐founded a start‐up company, WARD247 ApS, with the aim of pursuing the regulatory and commercial activities of the WARD‐project (Wireless Assessment of Respiratory and circulatory Distress, a project developing a clinical support system for continuous wireless monitoring of vital signs). WARD247 ApS has obtained licence agreement for any WARD‐project software and patents. One patent has been filed: “Wireless Assessment of Respiratory and circulatory Distress (WARD)—Clinical Support System (CSS)—an automated clinical support system to improve patient safety and outcomes”. Christian Sylvest Meyhoff reports in addition direct and indirect departmental research funding from Boehringer Ingelheim and Merck, Sharp & Dohme as well as lecture fees from Radiometer. Otherwise, no conflicts of interest.

## Supporting information


**Data S1** DEX‐2‐TKA trial

## References

[1] Lunn TH , Kristensen BB , Andersen L , et al. Effect of high‐dose preoperative methylprednisolone on pain and recovery after total knee arthroplasty: a randomized, placebo‐controlled trial. Br J Anaesth. 2011;106:230‐238. 21131371 10.1093/bja/aeq333

[2] Zhang JM , An J . Cytokines, inflammation, and pain. Int Anesthesiol Clin. 2007;45:27‐37. 17426506 10.1097/AIA.0b013e318034194e PMC2785020

[3] Holte K , Kehlet H . Perioperative single‐dose glucocorticoid administration: pathophysiologic effects and clinical implications.J. Am Coll Surg. 2002;195:694‐712. 10.1016/s1072-7515(02)01491-6 12437261

[4] Gasbjerg KS , Hägi‐Pedersen D , Haxholdt Lunn T , et al. Effect of dexamethasone as an analgesic adjuvant to multimodal pain treatment after total knee arthroplasty. BMJ. 2022;376:e067325. 34983775 10.1136/bmj-2021-067325 PMC8724786

[5] Ferreira SH , Cunha FQ , Lorenzetti BB , et al. Role of lipocortin‐1 in the anti‐hyperalgesic actions of dexamethasone. Br J Pharmacol. 1997;121:883‐888. 9222544 10.1038/sj.bjp.0701211 PMC1564768

[6] Romundstad L , Stubhaug A . Glucocorticoids for acute and persistent postoperative neuropathic pain: What is the evidence? Anesthesiology. 2007;107:371‐373. 17721239 10.1097/01.anes.0000279487.27940.5c

[7] Li H , Xie W , Strong JA , Zhang J‐M . Systemic anti‐inflammatory corticosteroid reduces mechanical pain behavior, sympathetic sprouting, and elevation of pro‐inflammatory cytokines in a rat model of neuropathic pain. Anesthesiology. 2007;107:469‐477. 17721250 10.1097/01.anes.0000278907.37774.8d PMC2174791

[8] Kisilewicz M , Rosenberg H , Vaillancourt C . Remifentanil for procedural sedation: a systematic review of the literature. Emerg Med J. 2017;34:294‐301. 28249938 10.1136/emermed-2016-206129

[9] Komatsu R , Turan AM , Orhan‐Sungur M , McGuire J , Radke OC , Apfel CC . Remifentanil for general anaesthesia: a systematic review. Anaesthesia. 2007;62:1266‐1280. 17991265 10.1111/j.1365-2044.2007.05221.x

[10] Fletcher D , Martinez V . Opioid‐induced hyperalgesia in patients after surgery: a systematic review and a meta‐analysis. Br J Anaesth. 2014;112:991‐1004. 24829420 10.1093/bja/aeu137

[11] Hogue CW , Bowdle TA , O'Leary C , et al. A multicenter evaluation of total intravenous anesthesia with remifentanil and propofol for elective inpatient surgery. Anesth Analg. 1996;83:279‐285. 8694306 10.1097/00000539-199608000-00014

[12] Gasbjerg KS , Hägi‐Pedersen D , Lunn TH , et al. DEX‐2‐TKA—DEXamethasone twice for pain treatment after Total knee arthroplasty: a protocol for a randomized, blinded, three‐group multicentre clinical trial. Acta Anaesthesiol Scand. 2020;64:267‐275. 31544230 10.1111/aas.13481

[13] Gasbjerg KS , Hägi‐Pedersen D , Lunn TH , et al. DEX‐2‐TKA ‐ DEXamethasone twice for pain treatment after total knee arthroplasty: detailed statistical analysis plan for a randomized, blinded, three‐group multicentre clinical trial. Acta Anaesthesiol Scand. 2020;64:839‐846. 32048274 10.1111/aas.13560

[14] Musizza B , Ribaric S . Monitoring the depth of anaesthesia. Sensors. 2010;10:10896‐10935. 22163504 10.3390/s101210896 PMC3231065

[15] American Society of Anesthesiologists . Practice advisory for intraoperative awareness and brain function monitoring. Anesthesiology. 2006;104:847‐864. 16571982 10.1097/00000542-200604000-00031

[16] Nørskov AK , Lange T , Nielsen EE , et al. Assessment of assumptions of statistical analysis methods in randomised clinical trials: the what and how. BMJ Evid Based Med 2021; 26: 121–6. 10.1136/bmjebm-2019-111268 31988195

[17] Effect of dexamethasone shortly before surgery on the intraoperative dose of remifentanil—full text view—ClinicalTrials.gov [Internet]. November 23, 2021. https://clinicaltrials.gov/ct2/show/NCT05002361

[18] Scott HB , Choi SW , Wong GTC , Irwin MG . The effect of remifentanil on propofol requirements to achieve loss of response to command vs. loss of response to pain. Anaesthesia. 2017;72:479‐487. 28094434 10.1111/anae.13781

[19] Herrera NA , Duchatsch F , Kahlke A , Amaral SL , Vasquez‐Vivar J . In vivo vascular rarefaction and hypertension induced by dexamethasone are related to phosphatase PTP1B activation not endothelial metabolic changes. Free Radic Biol Med. 2020;152:689‐696. 31978540 10.1016/j.freeradbiomed.2020.01.012 PMC8546799

[20] Fang F , Zhang Y , Tang J , et al. Association of corticosteroid treatment with outcomes in adult patients with sepsis: a systematic review and meta‐analysis. JAMA Intern Med. 2019;179:213. 30575845 10.1001/jamainternmed.2018.5849 PMC6439648

[21] Xu R , Wang Q , Huang Y , et al. Do low‐dose corticosteroids improve survival or shock reversal from septic shock in adults? Meta‐analysis with trial sequential analysis. J Int Med Res. 2018;46:2513‐2524. 29911468 10.1177/0300060518774985 PMC6124298

[22] Steinthorsdottir KJ , Kehlet H , Aasvang EK . Surgical stress response and the potential role of preoperative glucocorticoids on post‐anesthesia care unit recovery. Minerva Anestesiol. 2017;83:1324‐1331. 28607335 10.23736/S0375-9393.17.11878-X

[23] Salerno A , Hermann R . Efficacy and safety of steroid use for postoperative pain relief. Update and review of the medical literature. J Bone Joint Surg Am. 2006;88:1361‐1372. 16757774 10.2106/JBJS.D.03018

[24] Lopes MW , Leal RB , Guarnieri R , et al. A single high dose of dexamethasone affects the phosphorylation state of glutamate AMPA receptors in the human limbic system.Transl. Psychiatry. 2016;6:986. 10.1038/tp.2016.251 PMC5290343 27959333

[25] Brown ES . Effects of glucocorticoids on mood, memory, and the hippocampus: treatment and preventive therapy. Ann N Y Acad Sci. 2009;1179:41‐55. 19906231 10.1111/j.1749-6632.2009.04981.x

