# Supplementary material for: Effect of dexamethasone on intraoperative remifentanil dose in total knee arthroplasty surgery under general anaesthesia
Source: Acta Anaesthesiol Scand. 2022 Aug 4;66(9):1070–6. doi: 10.1111/aas.14118 (PMC9543467; doi:10.1111/aas.14118)
Supplement: Supplementary file 1 — Data S1 DEX‐2‐TKA trial [file AAS-66-1070-s001.pdf]

20 October 2021

DEX-2-TKA trial

Substudy analyses

Markus Harboe Olsen & Janus Christian Jakobsen

Markus Harboe Olsen (MHO) analysed the data at 20:06:09, 11 October 2021 and Janus Christian Jakobsen (JCJ) analysed them at 16:37:11, 15 October 2021.

Assumptions were tested for the pre-planned linear regression analyses. None of the outcomes fulfilled the assumptions, and we chose to use the corresponding non-parametric analyses. This includes either van Elteren for those where stratification for site was possible and Wilcoxon rank sum test without continuity correction for BIS where data was only available for one site. Furthermore, Hodges-Lehmann confidence intervals was used.

The results from the analyses carried out in R (by MHO) and in Stata (by JCJ) are not identical. The statistical analysis developed as part of the R-package was developed as 'an alternative to the van Elteren test' [1,2]. Thus, we replicated the van Elteren test from Stata in SAS (by MHO) and the results were identical.

MHO and JCJ agrees that the highlighted analyses in the Stata-report should be those reported in the final manuscript.

References

1. Kawaguchi, A., Koch, G. G., & Wang, X. (2011). Stratified multivariate Mann–Whitney estimators for the comparison of two treatments with randomization based covariance adjustment. Statistics in Biopharmaceutical Research, 3(2), 217-231.
2. Mehrotra, D. V., Lu, X., & Li, X. (2010). Rank-based analyses of stratified experiments: alternatives to the van Elteren test. The American Statistician, 64(2), 121-130.

The NPAR1WAY Procedure

Stratum Information		
Stratum	site_no	N Obs
1	1	31
2	2	28
3	3	29

Stratum Weights	Stratum Size
Ranks	Within Strata

Class Information		
Class	group	N Obs
1	1	28
2	0	60

Wilcoxon Scores by Strata for Ulti_inf Classified by group						
Stratum	N Obs	Class N Obs	Statistic	Expected	Std Dev	Mean
1	30	7	57.0	108.50	20.394035	8.142857
2	28	9	108.0	130.50	20.328551	12.000000
3	29	11	150.0	165.00	22.248595	13.636364

Stratified Wilcoxon (Van Elteren) Test for Ulti_inf Classified by group							
N Obs	N Strata	Statistic	Expected	Std Dev	Z	Pr < Z	Pr > |Z|
87	3	10.6	13.50	1.214156	-2.4191	0.0078	0.0156

The NPAR1WAY Procedure

Stratum Information		
**Stratum**	**site_no**	**N Obs**
**1**	1	31
**2**	2	28
**3**	3	29

**Stratum Weights**	Stratum Size
**Ranks**	Within Strata

Class Information		
**Class**	**group**	**N Obs**
**1**	1	28
**2**	0	60

Wilcoxon Scores by Strata for Prop_inf<br>Classified by group						
**Stratum**	**N Obs**	**Class N Obs**	**Statistic**	**Expected**	**Std Dev**	**Mean**
**1**	30	7	53.0	108.50	20.394035	7.571429
**2**	28	9	111.0	130.50	20.328551	12.333333
**3**	29	11	183.0	165.00	22.248595	16.636364

Stratified Wilcoxon (Van Elteren) Test for Prop_inf<br>Classified by group							
**N Obs**	**N Strata**	**Statistic**	**Expected**	**Std Dev**	**Z**	**Pr < Z**	**Pr > |Z|**
87	3	11.6	13.50	1.214156	-1.5342	0.0625	0.1250

The NPAR1WAY Procedure

Stratum Information		
Stratum	site_no	N Obs
1	1	31
2	2	28
3	3	29

Stratum Weights	Stratum Size
Ranks	Within Strata

Class Information		
Class	group	N Obs
1	1	28
2	0	60

Wilcoxon Scores by Strata for POA_time Classified by group						
Stratum	N Obs	Class N Obs	Statistic	Expected	Std Dev	Mean
1	30	8	119.00	124.00	21.318170	14.875000
2	28	9	102.00	130.50	20.328551	11.333333
3	29	11	170.50	165.00	22.243115	15.500000

Stratified Wilcoxon (Van Elteren) Test for POA_time Classified by group							
N Obs	N Strata	Statistic	Expected	Std Dev	Z	Pr < Z	Pr > |Z|
87	3	13.04	14.00	1.230454	-0.7808	0.2175	0.4349

```
------------------------------------------------------------------------------------------------------------------------
      name:  <unnamed>
       log:  /Users/janusjakobsen/Desktop/Active projects/Dex SubPubl/DEX_SUBP_RESULTS.log
  log type:  text
 opened on:  15 Oct 2021, 16:37:11

. *REMI

.
.
.
.
. by group_no, sort : summarize Ulti_inf, detail

------------------------------------------------------------------------------------------------------------------------
-> group_no = 0PBO

                          Ulti_inf
-------------------------------------------------------------
      Percentiles      Smallest
 1%     10.18072       10.18072
 5%     11.52461       11.52461
10%     12.08791       12.08791       Obs                  27
25%     14.93056       12.61364       Sum of wgt.          27

50%     20.46134                      Mean            20.2888
                        Largest       Std. dev.      5.803548
75%     25.39367       26.86004
90%     27.35562       27.35562       Variance       33.68116
95%         28.9           28.9       Skewness      -.1048162
99%      29.2973        29.2973       Kurtosis           1.72

------------------------------------------------------------------------------------------------------------------------
-> group_no = 1Dexa
```

```
                         Ulti_inf
-------------------------------------------------------------
      Percentiles      Smallest
 1%     5.917667       5.917667
 5%      13.6217         10.625
10%     15.93114       13.16288       Obs                  60
25%     19.14542       14.08053       Sum of wgt.          60

50%     22.71889                      Mean           23.67244
                        Largest       Std. dev.      7.268212
75%     27.68357       37.40343
90%     33.98183       39.28579       Variance        52.8269
95%     38.34461        40.0904       Skewness       .3701744
99%     40.45455       40.45455       Kurtosis       3.081101


.
. by group_no, sort : inspect Ulti_inf

--------------------------------------------------------------------------------------------------------------
--------------------
-> group_no = 0PBO

Ulti_inf:  Ulti_inf                              Number of observations
-------------------                    ---------------------------------------
                                             Total      Integers   Nonintegers
|      #       #               Negative          -             -             -
|      #       #   #           Zero              -             -             -
|      #       #   #           Positive         27             -            27
|  #   #       #   #                   -----------   -----------   -----------
|  #   #   #   #   #           Total            27             -            27
|  #   #   #   #   #           Missing           1
+----------------------                -----------
10.18072        29.2973                         28
  (27 unique values)

--------------------------------------------------------------------------------------------------------------
--------------------
```

```
-> group_no = 1Dexa

Ulti_inf:  Ulti_inf                                Number of observations
--------------------                   ---------------------------------------
                                                Total      Integers   Nonintegers
|          #                 Negative               -             -             -
|          #                 Zero                   -             -             -
|      #   #                 Positive              60             -            60
|      #   #                           -----------   -----------   -----------
|      #   #   #             Total                 60             -            60
|  .   #   #   #   #         Missing                -
+----------------------                -----------
5.917667       40.45455                            60
  (60 unique values)


.
. vanelteren Ulti_inf, by(group_no) strata(site_no)

Generalized Wilcoxon-Mann-Whitney Ranksum Test (van Elteren's Test)


                                                  Variance of
                       Weighted    Expected       Weighted
   Stratum     |    n    | Ranksum    | Ranksum   |     Ranksum
-------------+--------+------------+----------+-------------
           1 |   30   |    1.84    |    3.5    |     0.433
           2 |   28   |    3.72    |    4.5    |     0.491
           3 |   29   |    5.00    |    5.5    |     0.550
-------------+--------+------------+----------+-------------
    Sums      |   87   |   10.56    |   13.5    |     1.474

Asymptotic test statistic
z =  -2.419
Prob(Z > |z|) =  .0156

.
. cendif Ulti_inf, by(group_no) level(95)
```

```
Y-variable: Ulti_inf (Ulti_inf)
Grouped by: group_no (group)
Group numbers:

      group |      Freq.     Percent        Cum.
------------+-----------------------------------
       0PBO |         27       31.03       31.03
      1Dexa |         60       68.97      100.00
------------+-----------------------------------
      Total |         87      100.00
Transformation: Fisher's z
95% confidence interval(s) for percentile difference(s)
between values of Ulti_inf in first and second groups:
   Percent    Pctl_Dif     Minimum     Maximum
        50  -3.0535081  -6.1845869   .20195322

.
.
.
.
.
. *PROP

.
. by group_no, sort : summarize Prop_inf, detail

--------------------------------------------------------------------------------------------------------------
--------------------
-> group_no = 0PBO

                          Prop_inf
-------------------------------------------------------------
      Percentiles      Smallest
 1%     1.566265       1.566265
 5%      2.06292        2.06292
10%      2.15416        2.15416       Obs                  27
25%     3.231027       2.981293       Sum of wgt.          27
```

```
50%     3.699049                      Mean           3.565543
                        Largest       Std. dev.      .7565115
75%     3.970277          4.375
90%     4.520144       4.520144       Variance       .5723097
95%     4.593009       4.593009       Skewness      -.8701968
99%       4.7333         4.7333       Kurtosis       3.668035

-------------------------------------------------------------------------------------------------------------
--------------------
-> group_no = 1Dexa

                          Prop_inf
-------------------------------------------------------------
      Percentiles      Smallest
 1%     1.951158       1.951158
 5%     2.192386       1.972917
10%     2.553024       2.081681       Obs                  60
25%     3.332781       2.303091       Sum of wgt.          60

50%     3.930397                      Mean           4.009032
                        Largest       Std. dev.      1.115085
75%     4.644269       5.798291
90%     5.281421        5.84594       Variance       1.243414
95%     5.822115       6.377682       Skewness       .6770076
99%     7.927124       7.927124       Kurtosis       4.341061


.
. by group_no, sort : inspect Prop_inf

-------------------------------------------------------------------------------------------------------------
--------------------
-> group_no = 0PBO

Prop_inf:  Prop_inf                              Number of observations
-------------------                       ---------------------------------------
                                                Total      Integers   Nonintegers
|              #             Negative               -             -             -
```

```
|             #            Zero                -            -            -
|         #   #            Positive           27            -           27
|         #   #   #                   -----------  -----------  -----------
|         #   #   #        Total              27            -           27
|  #   .  #   #   #        Missing             1
+----------------------               -----------
1.566265         4.7333                       28
  (27 unique values)

----------------------------------------------------------------------------------------------------------------
--------------------
-> group_no = 1Dexa

Prop_inf:  Prop_inf                             Number of observations
-------------------                   ---------------------------------------
                                            Total      Integers   Nonintegers
|      #                   Negative            -            -            -
|      #                   Zero                -            -            -
|      #                   Positive           60            -           60
|      #   #                          -----------  -----------  -----------
|  #   #   #               Total              60            -           60
|  #   #   #   .   .       Missing             -
+----------------------               -----------
1.951158       7.927124                       60
  (60 unique values)


.
. vanelteren Prop_inf, by(group_no) strata(site_no)

Generalized Wilcoxon-Mann-Whitney Ranksum Test (van Elteren's Test)


                                                    Variance of
                       Weighted    Expected     Weighted
   Stratum    |   n    | Ranksum   | Ranksum  |   Ranksum
-------------+-------+-----------+----------+-------------
          1 |   30   |    1.71   |    3.5   |    0.433
```

```
          2 |   28  |    3.83    |    4.5    |    0.491
          3 |   29  |    6.10    |    5.5    |    0.550
-------------+-------+------------+-----------+-------------
    Sums     |   87  |   11.64    |   13.5    |    1.474

Asymptotic test statistic
z =  -1.534
Prob(Z > |z|) =   .125

.
. cendif Prop_inf, by(group_no) level(95)
Y-variable: Prop_inf (Prop_inf)
Grouped by: group_no (group)
Group numbers:

      group |      Freq.     Percent        Cum.
------------+-----------------------------------
       0PBO |         27       31.03       31.03
      1Dexa |         60       68.97      100.00
------------+-----------------------------------
      Total |         87      100.00
Transformation: Fisher's z
95% confidence interval(s) for percentile difference(s)
between values of Prop_inf in first and second groups:
   Percent     Pctl_Dif      Minimum      Maximum
        50   -.33226882   -.72416667    .04404329

.
.
.
.
. *BIS

. by group_no, sort : summarize BIS, detail

--------------------------------------------------------------------------------------------------------------
--------------------
-> group_no = 0PBO
```

```
                             BIS
-------------------------------------------------------------
      Percentiles      Smallest
 1%        40.46          40.46
 5%        40.46          42.27
10%        42.27          42.58       Obs                  11
25%        42.58          43.55       Sum of wgt.          11

50%        44.67                      Mean           49.00727
                        Largest       Std. dev.       7.48373
75%        55.46          53.24
90%        58.98          55.46       Variance       56.00622
95%         61.8          58.98       Skewness       .4776936
99%         61.8           61.8       Kurtosis       1.739342

--------------------------------------------------------------------------------------------------------------
--------------------
-> group_no = 1Dexa

                             BIS
-------------------------------------------------------------
      Percentiles      Smallest
 1%         35.2           35.2
 5%         35.2           38.2
10%         38.2             39       Obs                  18
25%        40.47          39.07       Sum of wgt.          18

50%        48.01                      Mean           48.17889
                        Largest       Std. dev.        8.7392
75%        55.16          55.54
90%        61.75          59.46       Variance       76.37361
95%        64.26          61.75       Skewness         .30987
99%        64.26          64.26       Kurtosis       1.960911


.
. by group_no, sort : inspect BIS
```

```
----------------------------------------------------------------------------------------------------------------
--------------------
-> group_no = 0PBO

BIS:  BIS                                      Number of observations
---------                             ---------------------------------------
                                                  Total      Integers   Nonintegers
|  #                             Negative             -             -             -
|  #                             Zero                 -             -             -
|  #                             Positive            11             -            11
|  #                                        -----------   -----------   -----------
|  #       #       #             Total               11             -            11
|  #   .   #   #   #             Missing             17
+-----------------------                    -----------
40.46              61.8                              28
  (11 unique values)

----------------------------------------------------------------------------------------------------------------
--------------------
-> group_no = 1Dexa

BIS:  BIS                                      Number of observations
---------                             ---------------------------------------
                                                  Total      Integers   Nonintegers
|  #                             Negative             -             -             -
|  #                             Zero                 -             -             -
|  #       #                     Positive            18             1            17
|  #       #   #   #                        -----------   -----------   -----------
|  #   #   #   #   #             Total               18             1            17
|  #   #   #   #   #             Missing             42
+-----------------------                    -----------
35.2              64.26                              60
  (18 unique values)


.
. vanelteren BIS, by(group_no) strata(site_no)
```

```
Generalized Wilcoxon-Mann-Whitney Ranksum Test (van Elteren's Test)


                                                          Variance of
                       Weighted     Expected       Weighted
   Stratum    |    n    | Ranksum    | Ranksum   |    Ranksum
-------------+-------+-----------+----------+-------------
          3 |   29  |     5.77   |     5.5   |     0.550
-------------+-------+-----------+----------+-------------
    Sums      |   29  |     5.77   |     5.5   |     0.550

Asymptotic test statistic
z =    .3596
Prob(Z > |z|) =  .7192

.
. cendif BIS, by(group_no) level(95)
Y-variable: BIS (BIS)
Grouped by: group_no (group)
Group numbers:

      group |      Freq.     Percent        Cum.
------------+-----------------------------------
       0PBO |         11       37.93       37.93
      1Dexa |         18       62.07      100.00
------------+-----------------------------------
      Total |         29      100.00
Transformation: Fisher's z
95% confidence interval(s) for percentile difference(s)
between values of BIS in first and second groups:
   Percent     Pctl_Dif      Minimum      Maximum
        50          1.6        -5.28         6.47

.
.
.
.
```

```
.
. *TIME SPENT

.
. by group_no, sort : summarize POA_time, detail

-----------------------------------------------------------------------------------------------------------
--------------------
-> group_no = 0PBO

                          POA_time
-------------------------------------------------------------
      Percentiles      Smallest
 1%            1              1
 5%            1              1
10%     1.033333       1.033333       Obs                  28
25%        1.475           1.25       Sum of wgt.          28

50%     1.858333                      Mean           2.189881
                        Largest       Std. dev.      .9656374
75%     3.041667            3.5
90%     3.616667       3.616667       Variance       .9324555
95%     3.683333       3.683333       Skewness       .7113845
99%          4.5            4.5       Kurtosis       2.412958

-----------------------------------------------------------------------------------------------------------
--------------------
-> group_no = 1Dexa

                          POA_time
-------------------------------------------------------------
      Percentiles      Smallest
 1%     .7833333       .7833333
 5%     1.133333            1.1
10%     1.233333       1.133333       Obs                  59
25%     1.466667           1.15       Sum of wgt.          59

50%         2.25                      Mean           2.389266
```

```
                           Largest       Std. dev.      1.047103
75%     2.966667       4.233333
90%     3.866667       4.366667       Variance       1.096425
95%     4.366667       5.233333       Skewness       .7924351
99%     5.383333       5.383333       Kurtosis         3.3001


.
. by group_no, sort : inspect POA_time

--------------------------------------------------------------------------------------------------------------
--------------------
-> group_no = 0PBO

POA_time:  POA_time                             Number of observations
-------------------                    ---------------------------------------
                                             Total      Integers   Nonintegers
|  #                          Negative           -             -             -
|  #                          Zero               -             -             -
|  #                          Positive          28             4            24
|  #   #                               -----------   -----------   -----------
|  #   #   #   #                Total             28             4            24
|  #   #   #   #   .          Missing            -
+----------------------                -----------
1                    4.5                        28
  (25 unique values)

--------------------------------------------------------------------------------------------------------------
--------------------
-> group_no = 1Dexa

POA_time:  POA_time                             Number of observations
-------------------                    ---------------------------------------
                                             Total      Integers   Nonintegers
|  #                          Negative           -             -             -
|  #                          Zero               -             -             -
|  #   #   #                  Positive          59             -            59
|  #   #   #                           -----------   -----------   -----------
```

```
|  #   #   #                 Total              59              -              59
|  #   #   #   #   .         Missing             1
+----------------------                -----------
.7833333       5.383333                         60
  (53 unique values)


.
. vanelteren POA_time, by(group_no) strata(site_no)

Generalized Wilcoxon-Mann-Whitney Ranksum Test (van Elteren's Test)


                                                     Variance of
                       Weighted    Expected       Weighted
   Stratum     |    n    | Ranksum    | Ranksum   |    Ranksum
-------------+-------+-----------+----------+-------------
           1 |   30   |    3.84    |    4.0    |     0.473
           2 |   28   |    3.52    |    4.5    |     0.491
           3 |   29   |    5.68    |    5.5    |     0.550
-------------+-------+-----------+----------+-------------
    Sums     |   87   |   13.04    |   14.0    |     1.514

Asymptotic test statistic
z =  -.7808
Prob(Z > |z|) =  .4349

.
. cendif POA_time, by(group_no) level(95)
Y-variable: POA_time (POA_time)
Grouped by: group_no (group)
Group numbers:

      group |      Freq.     Percent        Cum.
------------+-----------------------------------
       0PBO |         28       32.18       32.18
      1Dexa |         59       67.82      100.00
------------+-----------------------------------
```

```
      Total |         87      100.00
Transformation: Fisher's z
95% confidence interval(s) for percentile difference(s)
between values of POA_time in first and second groups:
   Percent    Pctl_Dif     Minimum     Maximum
        50  -.18333333  -.63333333   .26666667

.
.
.
.
.
. log close
      name:  <unnamed>
       log:  /Users/janusjakobsen/Desktop/Active projects/Dex SubPubl/DEX_SUBP_RESULTS.log
  log type:  text
 closed on:  15 Oct 2021, 16:37:30
--------------------------------------------------------------------------------------------------------------
--------------------
```

DEX2TKA substudy analyses

Markus Harboe Olsen

2021-11-05 20:25:08

Contents

Characteristics **2**

Infusion rate of Ultiva **4**

Infusion rate of Propofol **5**

BIS **6**

POA time **7**

Characteristics

```
##                          Stratified by group
##                           0              1
##   n                            60             28
##   site (%)
##      Aggregated site           23 (38.3)       8 (28.6)
##      Bispebjerg Hospital       19 (31.7)       9 (32.1)
##      Næstved Sygehus           18 (30.0)      11 (39.3)
##   Ulti_inf (mean (SD))      23.67 (7.27)   20.29 (5.80)
##   Prop_inf (mean (SD))       4.01 (1.12)    3.57 (0.76)
##   BIS (mean (SD))           48.18 (8.74)   49.01 (7.48)
##   POA_time (mean (SD))     143.36 (62.83) 131.39 (57.94)
```

```
##
##      ### Summary of continuous variables ###
##
## group: 0
##           n miss p.miss mean sd median p25 p75 min max skew kurt
## Ulti_inf 60    0      0   24  7     23  19  28   6  40  0.4  0.2
## Prop_inf 60    0      0    4  1      4   3   5   2   8  0.7  1.6
## BIS      60   42     70   48  9     48  41  55  35  64  0.3 -1.0
## POA_time 60    1      2  143 63    135  90 176  47 323  0.8  0.4
## ---------------------------------------------------------------
## group: 1
##           n miss p.miss mean   sd median p25 p75 min max skew kurt
## Ulti_inf 28    1      4   20  5.8     20  16  25  10  29 -0.1 -1.3
## Prop_inf 28    1      4    4  0.8      4   3   4   2   5 -0.9  1.1
## BIS      28   17     61   49  7.5     45  43  54  40  62  0.6 -1.3
## POA_time 28    0      0  131 57.9    111  92 181  60 270  0.8 -0.5
##
## Standardize mean differences
##             1 vs 2
## Ulti_inf 0.5144828
## Prop_inf 0.4654502
## BIS      0.1018206
## POA_time 0.1979604
##
## ========================================================================================
##
##      ### Summary of categorical variables ###
##
## group: 0
##   var  n miss p.miss               level freq percent cum.percent
##  site 60    0    0.0     Aggregated site   23    38.3        38.3
##                      Bispebjerg Hospital   19    31.7        70.0
##                          Næstved Sygehus   18    30.0       100.0
##
## ---------------------------------------------------------------
## group: 1
##   var  n miss p.miss               level freq percent cum.percent
##  site 28    0    0.0     Aggregated site    8    28.6        28.6
##                      Bispebjerg Hospital    9    32.1        60.7
##                          Næstved Sygehus   11    39.3       100.0
##
##
## Standardize mean differences
##         1 vs 2
## site 0.2327469
```

Infusion rate of Ultiva

```
## [1] "VanElteren"

## Call:
## sanon.formula(formula = Ulti_inf ~ grp(group) + strt(site), data = df)
##
##
##          Estimate Std.Err Chisq Pr(>Chisq)
## Ulti_inf  -0.1660  0.0633  6.88     0.0087 **
## ---
## Signif. codes:  0 '***' 0.001 '**' 0.01 '*' 0.05 '.' 0.1 ' ' 1
## Note that the estimates of responses are for the (MW estimate - 0.5).

## [1] "HodgesLehmann"

##        est     lwr.ci     upr.ci
##  3.0462742 -0.2626821  6.2454017
```

Infusion rate of Propofol

```
## [1] "VanElteren"
```

```
## Call:
## sanon.formula(formula = Prop_inf ~ grp(group) + strt(site), data = df)
##
##
##          Estimate Std.Err Chisq Pr(>Chisq)
## Prop_inf  -0.1053  0.0737  2.04       0.15
## Note that the estimates of responses are for the (MW estimate - 0.5).
```

```
## [1] "HodgesLehmann"
```

```
##         est      lwr.ci      upr.ci
##  0.33233491 -0.07349859  0.76451133
```

BIS

```
## [1] "Wilcox"
```

```
##
##  Wilcoxon rank sum exact test
##
## data:  BIS by factor(group)
## W = 91, p-value = 0.7402
## alternative hypothesis: true location shift is not equal to 0
```

```
## [1] "HodgesLehmann"
```

```
##    est lwr.ci upr.ci
##  -1.60  -6.76   5.89
```

POA time

```
## [1] "VanElteren"
```

```
## Call:
## sanon.formula(formula = POA_time ~ grp(group) + strt(site), data = df)
##
##
##          Estimate Std.Err Chisq Pr(>Chisq)
## POA_time  -0.0538  0.0707  0.58       0.45
## Note that the estimates of responses are for the (MW estimate - 0.5).
```

```
## [1] "HodgesLehmann"
```

```
##       est    lwr.ci    upr.ci
##  10.99996 -16.99991  38.00000
```
